# Comparative Studies on Behavioral, Cognitive and Biomolecular Profiling of ICR, C57BL/6 and Its Sub-Strains Suitable for Scopolamine-Induced Amnesic Models

**DOI:** 10.3390/ijms18081735

**Published:** 2017-08-09

**Authors:** Govindarajan Karthivashan, Shin-Young Park, Joon-Soo Kim, Duk-Yeon Cho, Palanivel Ganesan, Dong-Kug Choi

**Affiliations:** 1 Department of Biotechnology, College of Biomedical and Health Science, Konkuk University, Chungju 27478, Korea; karthivashan@gmail.com (G.K.); ifresha@nate.com (S.-Y.P.); kgfdkr@gmail.com (J.-S.K.); ejrdus1026@naver.com (D.-Y.C.); palanivel67@gmail.com (P.G.); 2 Nanotechnology Research Center, Department of Applied Life Science, College of Biomedical and Health Science, Konkuk University, Chungju 27478, Korea

**Keywords:** scopolamine, amnesia, cognition, CREB/BDNF, outbred ICR strains, inbred C57BL/6 strains, C57BL/6N, C57BL/6J sub-strains

## Abstract

Cognitive impairment and behavioral disparities are the distinctive baseline features to investigate in most animal models of neurodegenerative disease. However, neuronal complications are multifactorial and demand a suitable animal model to investigate their underlying basal mechanisms. By contrast, the numerous existing neurodegenerative studies have utilized various animal strains, leading to factual disparity. Choosing an optimal mouse strain for preliminary assessment of neuronal complications is therefore imperative. In this study, we systematically compared the behavioral, cognitive, cholinergic, and inflammatory impairments of outbred ICR and inbred C57BL/6 mice strains subject to scopolamine-induced amnesia. We then extended this study to the sub-strains C57BL/6N and C57BL/6J, where in addition to the above-mentioned parameters, their endogenous antioxidant levels and cAMP response-element binding protein (CREB)/brain-derived neurotrophic factor (BDNF) protein expression were also evaluated. Compared with the ICR strain, the scopolamine-inflicted C57BL/6 strains exhibited a substantial reduction of spontaneous alternation and an approximately two-fold increase in inflammatory protein expression, compared to the control group. Among the sub-strains, scopolamine-treated C57BL/6N strains exhibited declined step-through latency, elevated acetylcholinesterase (AChE) activity and inflammatory protein expression, associated with reduced endogenous antioxidant levels and p-CREB/BDNF expression, compared to the control and tacrine-treated groups. This indicates that the C57BL/6N strains exhibit significantly enhanced scopolamine-induced neuronal impairment compared to the other evaluated strains.

## 1. Introduction

Neurodegenerative diseases can be defined as genetic and erratic conditions characterized by progressive nervous system dysfunction. The prevalence of aging-related neurodegenerative diseases has been anticipated to drastically elevate in the near future [1,2]. Alzheimer’s disease (AD) is the most commonly scrutinized neurodegenerative model, as its prevalence is expected to increase radically in developing countries over the next 40 years [3]. AD involves the selective degeneration of cholinergic neurons, which innervate hippocampal and interrelated parts, thereby affecting cognitive functions, especially memory [4,5]. Cognitive impairments (i.e., anterograde amnesia), behavioral disparities, neurofibrillary changes in medial temporal lobe structures (e.g., the hippocampus and entorhinal cortex), and mediated neuronal cell death are the clinical hallmarks of AD pathology, contributing to the staging and tracking of disease [6]. Over the last decade, scientific advances such as computer-aided drug design yielded a surplus of new drug candidates, but a declining success rate was habitually observed during clinical development [7,8]. One of the most significant tasks is the selection of an appropriate animal model during preclinical screening investigations [9]. The act of picking a precise mouse strain is crucial, as the neurodegenerative disorders involve several interlinked molecular pathways, hence demanding suitable animal strains to investigate their underlying mechanism. Cognitive decline, behavioral disparities, region-specific neuronal loss, oxidative stress, and neuroinflammation are common paradigms for most of the neurodegenerative disorders instigated by ageing or drugs [10,11,12]. Additionally, accumulating evidence suggests the crucial role of the cAMP response-element binding protein (CREB)/brain-derived neurotrophic factor (BDNF) pathway, which has been associated with numerous neurodegenerative complications [13,14,15]. These aspects of the clinical phenotype should be considered when choosing the animal strain for a particular model of disease. On the contrary, numerous strains express diverse genetic manipulation and may not be suitable for behavioral and cognitive studies. Thus, selecting the optimal animal model for studying the baseline factors of neurodegenerative diseases is essential. In this study, we compared the commonly used inbred C57BL/6 mouse strain with the outbred ICR mouse strain to rationalize the effect of genetic variation. While abundant scientific research has used the C57BL/6 mouse strain as a general model for neurodegenerative disorders [16,17,18], continued breeding eventually results in the development of two genetically-distinct sub-strains: the C57BL/6J (Jackson Laboratory) and the C57BL/6N (National Institute of Health). Thus, we extended our study to compare these two sub-strains in order to obtain a clearer picture of behavioral and cognitive stability. The scopolamine-induced amnesia model is a well-established and commonly practiced disease model to parameterize behavioral and cognitive impairment [19,20]. Thus, the same model has been adapted in this study, with tacrine (a noncompetitive acetylcholinesterase (AChE) inhibitor which is used in the treatment of AD complication) as a positive control [21,22]. Although numerous studies have been reported on the strain comparisons based on genetic difference, rudimentary behavioral facets (i.e., rearing and exploration), and fear responses [23,24,25], only a few studies have examined more intricate behavioral inflections—especially with regard to the ICR and C57BL/6 mouse strains. Therefore, in this study, we initially screened the inbred and outbred mouse strains based on exploratory behavior toward new environments (i.e., Y-Maze spontaneous alternation), a learning and memory task (i.e., passive avoidance test), and variation in cholinergic system (acetylcholinesterase activity) at the hippocampus. In addition to the above-discussed parameters, we comparatively evaluated the sub-strains C57BL/6N and C57BL/6J for modulations associated with cognitive functions such as inflammatory protein expression, endogenous antioxidant levels, and CREB/BDNF protein expression at the hippocampus. This study delivers guidance on determining suitable strains, paving the way for analogous scientific investigations into biological mechanisms involved in scopolamine-induced amnesia with possible relevance to major neurodegenerative disorders.

## 2. Results

### 2.1. Modulations on Spatial Learning and Memory Deficits

**Figure 1:**
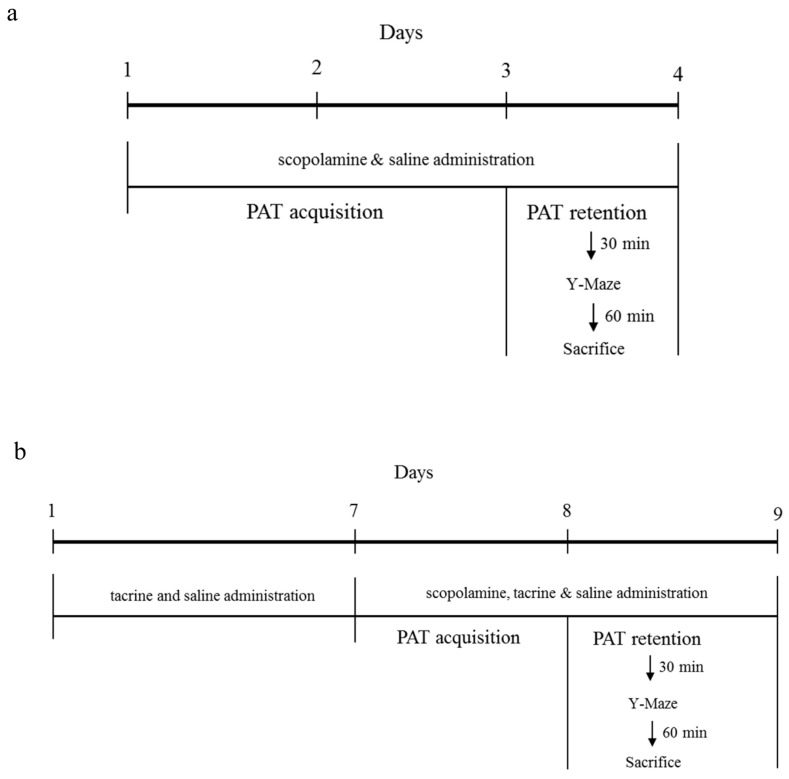
Animal experimental design: (**a**) Comparison of outbred ICR and inbred C57BL/6 strain; (**b**) Comparison of C57BL/6 substrains-C57BL/6N and C57BL/6J. PAT: passive avoidance test.

The animal experimental study design of ICR Vs C57BL/6 strain comparison and C57BL/6N Vs C57BL/6J sub-strain comparison, suitable for scopolamine induced animal model is depicted in Figure 1a,b respectively. To comparatively evaluate the behavioral deficits inflicted by scopolamine the among the described strains, we adapted step-through passive avoidance test (PAT) and spontaneous alternation performance (Y-Maze) test.

#### 2.1.1. ICR and C57BL/6 Comparison Study

**Figure 2:**
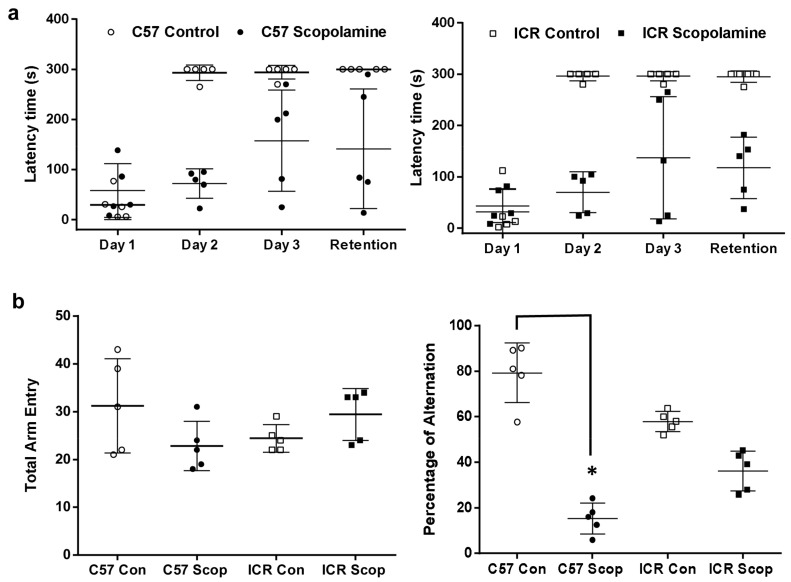
Spatial learning and memory analysis comparison of ICR and C57BL/6 strains in scopolamine-induced amnesic model: (**a**) Step-through passive avoidance test—latency variance of the 3-day acquisition and fourth day retention trial; (**b**) Spontaneous alternation performance (Y-maze test). Data are expressed as mean ± SD (*n* = 5). The PAT data was analyzed using two-way ANOVA—Tukey’s multiple comparison test, # *p* < 0.05, within the same strains when the control and scopolamine treated groups are significant; Y-Maze data was analyzed using one-way ANOVA-Tukey’s multiple comparison test, * *p* < 0.05, compared with the control group.

As seen in Figure 2a, the step-through latency of the C57BL/6 control group was significantly increased and retained over the daily course of acquisition-tasking compared to the scopolamine group. Alternatively, the step-through latency of the ICR control group gradually increased over the daily course of acquisition-tasking. On the second day, the scopolamine-administered group in both mouse strains (C57BL/6 and ICR) expressed a significant (F (4, 60) = 1.184; *p* < 0.05) reduction in step-through latency, with values of 72 ± 29 s and 70 ± 40 s, compared to 293 ± 16 s and 296 ± 9 s in the respective control groups. Subsequently, on the fourth day (retention-tasking), the control group for both strains (ICR and C57BL/6) exhibited significantly (F (4, 60) = 1.184; *p* < 0.05) higher latency time compared to the scopolamine-administered groups. In the Y-Maze test (Figure 2b), the scopolamine-treated group of the C57BL/6 strain exhibited significantly (F (3, 16) = 48.37; *p* < 0.05) lower percentage of spontaneous alternation, with a value of 15 ± 6.75% compared to the control group at 79 ± 11.73%. In contrast, the scopolamine-treated and control groups of the ICR strain exhibited a non-significant difference in spontaneous alternation percentage. In addition, no significant differences were observed among the groups of both strains C57BL/6 and ICR in terms of total arm entries with the values of control (31 ± 10 n and 24 ± 3 n) and scopolamine (23 ± 5 n and 29 ± 5 n), respectively.

#### 2.1.2. C57BL/6N and C57BL/6J Comparison Study

**Figure 3:**
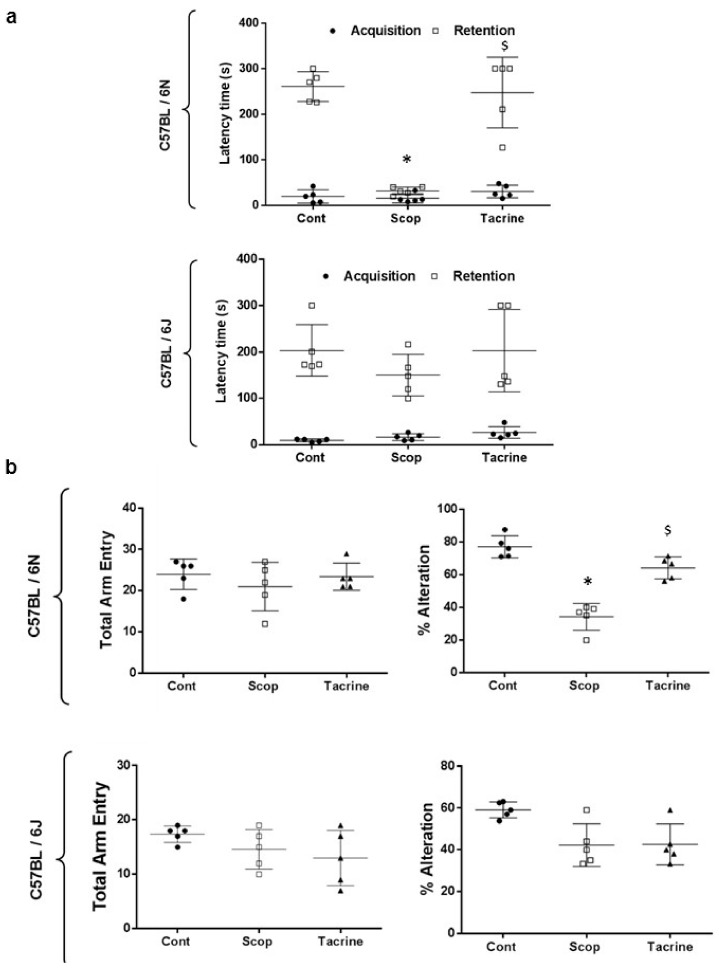
Spatial learning and memory analysis comparison of C57BL/6 substrains—C57BL/6N and C57BL/6J in scopolamine-induced amnesic model: (**a**) Step-through passive avoidance test—latency time variance during acquisition and retention; (**b**) Spontaneous alternation performance (Y-Maze test)—number of arm entries and percentage of alternation (circle dots—control group; square—scopolamine group; triangle represents tacrine group). Tacrine (10 mg/kg) was used as positive control. Data are expressed as mean ± SD (*n* = 5). One-way ANOVA-Tukey’s multiple comparison test was performed where, * *p* < 0.05 scopolamine compared with the control group; ^$^ *p* < 0.05 tacrine compared with scopolamine-treated group.

From Figure 3a, during the acquisition session, the latency time acquired from both sub-strains 6N and 6J did not significantly differ among the treated groups. Subsequently, during the retention test, the scopolamine-treated group of the 6N strain exhibited a significantly (F (4, 20) = 1.556; *p* < 0.05) reduced step-through latency time of 32 ± 9 s, compared to the control (261 ± 33 s) and tacrine (248 ± 78 s) groups. In contrast, the step-through latency time of the 6J strain did not significantly differ among the scopolamine, control, and tacrine-treated groups.

The Y-Maze test results shown in from Figure 3b reveal that the scopolamine-treated group in both the 6N and 6J sub-strains exhibited lower percentages of spontaneous alternation, with values of 34.22 ± 8.18% and 42.26 ± 10.25%, than the control group, with respective values of 77.02 ± 6.77% and 59.07 ± 3.84%; however, only 6N showed significant (F (5, 24) = 21.06; *p* < 0.05) difference. Additionally, no significant differences were observed between the control and tacrine-treated groups in terms of spontaneous alternation. The tacrine-treated group for 6N presented a significantly (F (5, 24) = 21.06; *p* < 0.05) higher percentage of spontaneous alternation (64.10 ± 6.74%) compared to the scopolamine-treated group (34.22 ± 8.18%), whereas no significant difference was observed between tacrine- and scopolamine-treated groups of the 6J strain. In addition, no significant differences were observed among the treated groups of both sub-strains C57BL/6N and C57BL/6J in terms of total arm entries.

### 2.2. AChE Activity

#### 2.2.1. ICR and C57BL/6 Comparison Study

**Figure 4:**
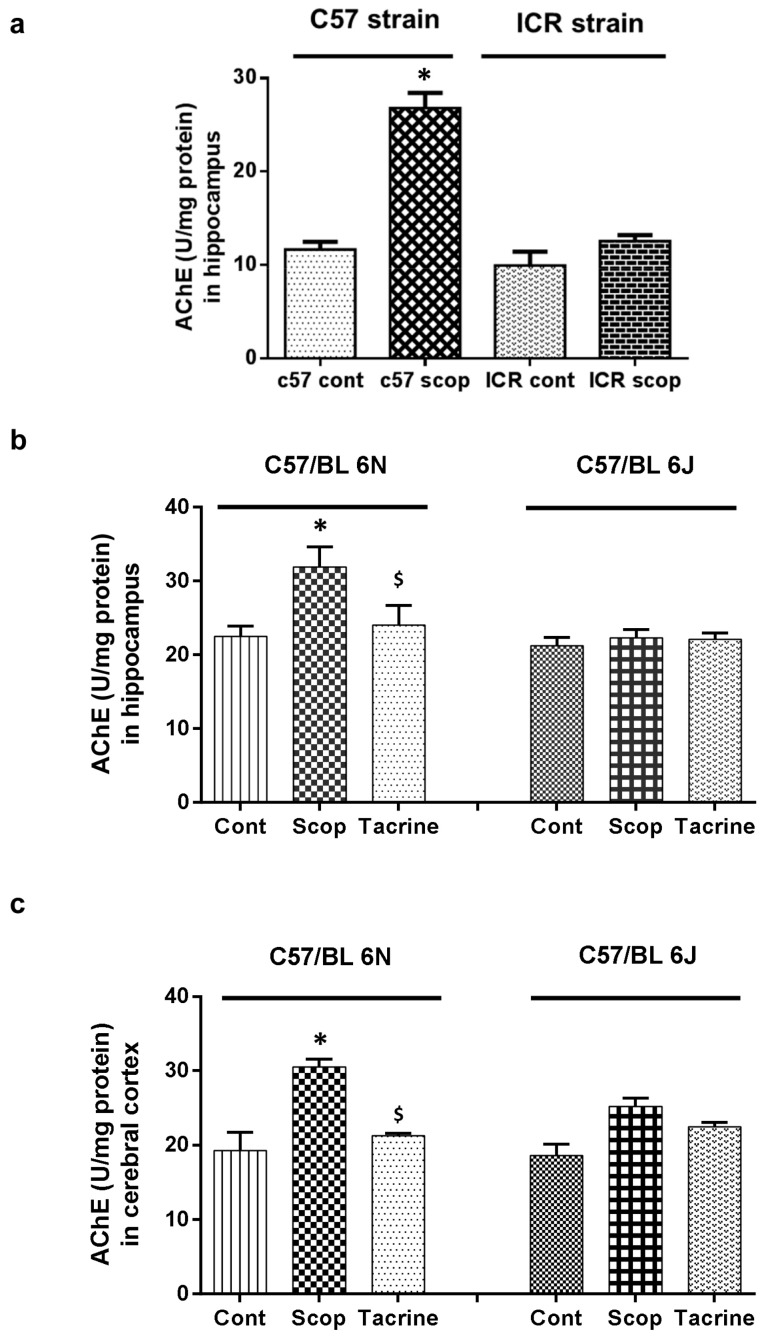
Acetylcholinesterase (AChE) activity in brain tissues of scopolamine-induced amnesic models: (**a**) Hippocampus of ICR and C57BL/6 strains; (**b**) Hippocampus and (**c**) cerebral cortex of C57BL/6N and C57BL/6J substrains. Data are expressed in U/mg protein as mean ± SD (*n* = 4). One-way ANOVA-Tukey’s multiple comparison test was performed where, * *p* < 0.05 scopolamine compared with the control group; ^$^ *p* < 0.05 tacrine compared with scopolamine-treated group.

In the C57BL/6 strain (Figure 4a), the hippocampus of the scopolamine-treated group exhibited significantly (F (3, 12) = 93.69; *p* < 0.05) higher AChE activity of 26.77 ± 1.64 U/mg protein, compared to the control group (11.64 ± 0.81 U/mg protein), whereas no significant difference was observed among these groups in the ICR strain.

#### 2.2.2. C57BL/6N and C57BL/6J Comparison Study

In the 6N strain (Figure 4b,c), the scopolamine-treated group exhibited significantly (F (5, 18) = 27.88; *p* < 0.05) higher AChE activity (31.8 ± 2.76 U/mg protein and 30.48 ± 1.06 U/mg protein) compared to the control (22.48 ± 1.40 U/mg protein and 19.30 ± 2.37 U/mg protein) and tacrine-treated (24.00 ± 2.71 U/mg protein and 21.25 ± 0.32 U/mg protein) groups, in both hippocampus and cerebral cortex, respectively. On the contrary, no significant difference in AChE activity was observed in either the hippocampus or cerebral cortex of the treated 6J groups.

### 2.3. Inflammatory Protein Expressions at Hippocampus

#### 2.3.1. ICR and C57BL/6 Comparison Study

**Figure 5:**
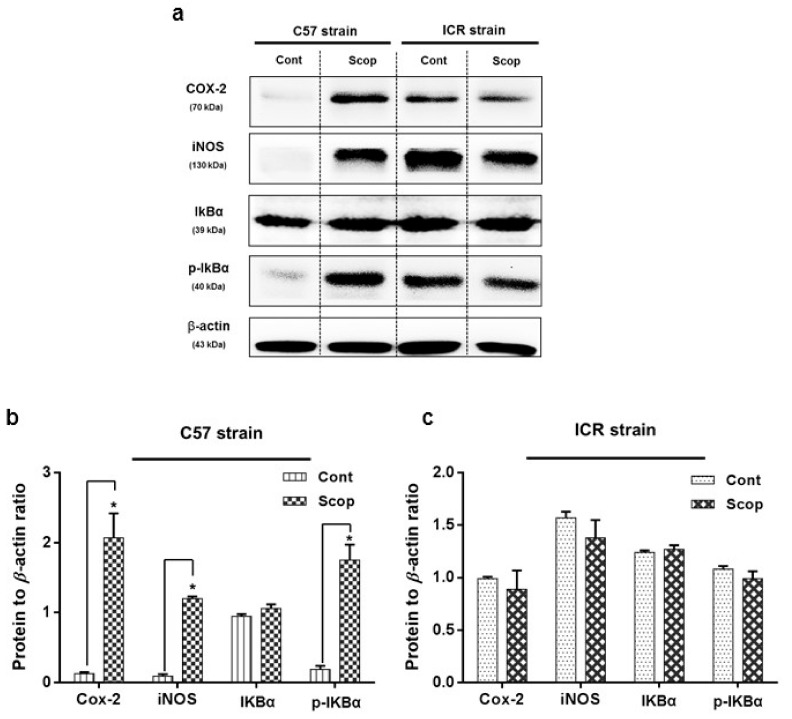
Inflammatory protein expression in hippocampus of scopolamine-induced amnesic models—ICR and C57BL/6 strains: (**a**) inducible nitric oxide synthase (iNOS), cyclooxygenase-2 (COX-2), nuclear factor of κ inhibitor α (IκBα) and phosphorylated-nuclear factor of κ inhibitor (p-IκBα) levels in the hippocampus determined by Western blotting. Quantification of inflammatory protein expression relative to β-actin in hippocampus of (**b**) C57BL/6 and (**c**) ICR strains. Data are expressed as mean ± SD (*n* = 3). One-way ANOVA-Tukey’s multiple comparison test was performed where, * *p* < 0.05 scopolamine compared with the control group.

In the C57BL/6 strain (Figure 5a,b and Figures S1–S5), the hippocampus of the scopolamine-treated group exhibited significantly (F (3, 8) = 42.55; F (3, 8) = 95.54; F (3, 8) = 130.03; *p* < 0.05) higher levels of inflammatory protein expression, with an approximately two-fold increase in cyclooxygenase-2 (COX-2) and phosphorylated—nuclear factor of κ inhibitor (p-IκBα) levels, and a one-fold increase in inducible nitric oxide synthase (iNOS) levels compare to the control group respectively, whereas the inflammatory protein expressions in the ICR strain (Figure 5a,c) did not significantly differ among the control and scopolamine-treated groups.

#### 2.3.2. C57BL/6N and C57BL/6J Comparison Study

**Figure 6:**
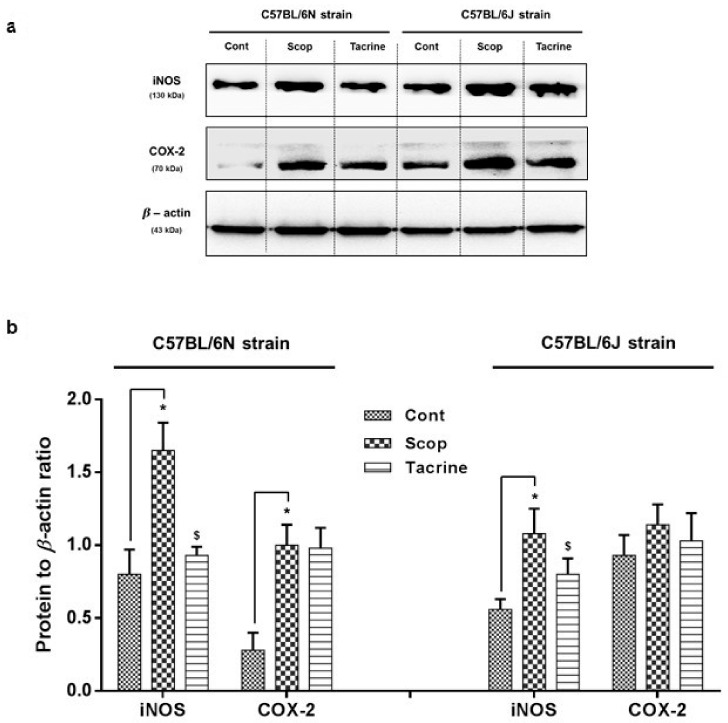
Inflammatory protein markers (iNOS and COX-2) expression in the hippocampus of scopolamine-induced amnesic models—C57BL/6N and C57BL/6J substrains: (**a**) Western blotting of inflammatory protein expression and (**b**) its quantification relative to β-actin expression. Data are expressed as mean ± SD (*n* = 3). One-way ANOVA-Tukey’s multiple comparison test was performed where, * *p* < 0.05 scopolamine compared with the control group; ^$^ *p* < 0.05 tacrine compared with scopolamine-treated group.

Among the C57BL/6 sub-strains, the scopolamine-treated group of the 6N strain (Figure 6, Figures S6 and S7) exhibited significantly (F (2, 8) = 27.18; F (2, 8) = 33.11; *p* < 0.05) higher levels of inflammatory protein expression, with an approximately two-fold increase in COX-2 and iNOS levels compared to the control group, which was substantially reversed in the tacrine-treated group. In the 6J strain, the scopolamine-treated group exhibited a significantly higher level of iNOS protein expression compared to the control group, but mild insignificant differences in COX-2 levels were observed among the treated groups.

### 2.4. Lipid Peroxidation Activity in the Hippocampus and Cerebral Cortex

**Figure 7:**
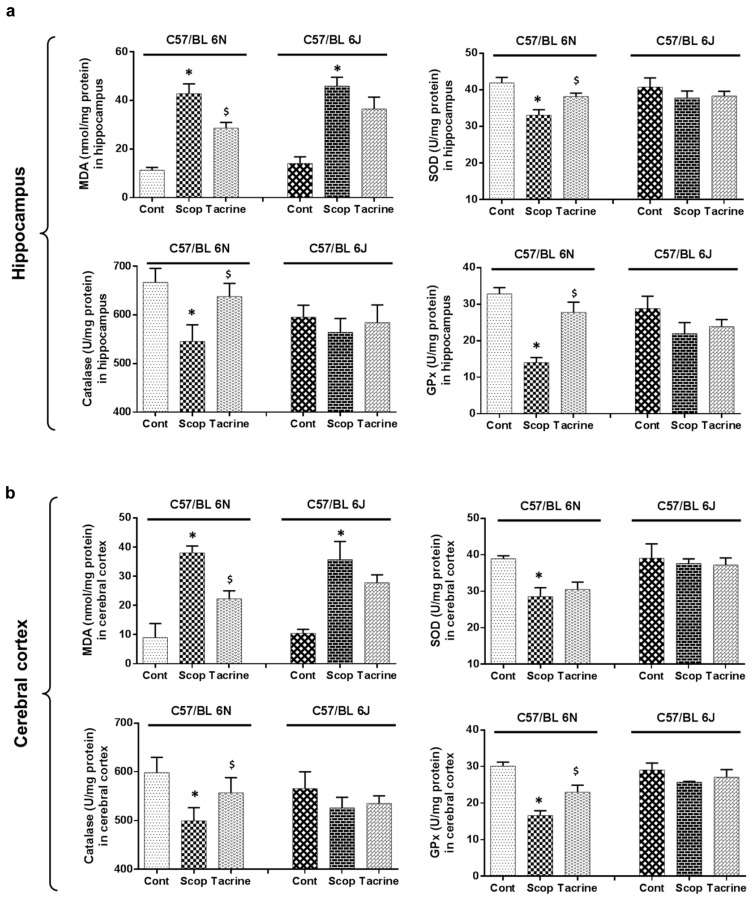
Endogenous level of oxidative stress biomarkers in brain tissue of scopolamine-induced amnesic models—C57BL/6N and C57BL/6J substrains: The lipid peroxidation (malondialdehyde, MDA) and antioxidant biomarkers (superoxide dismutase, SOD, catalase, CAT, glutathione peroxidase, GPx) levels in the (**a**) hippocampus and (**b**) cerebral cortex of mice were determined. Data are expressed as mean ± SD (*n* = 4; pooled biological replications). One-way ANOVA-Tukey’s multiple comparison test was performed where, * *p* < 0.05 scopolamine compared with the control group; ^$^ *p* < 0.05 tacrine compared with scopolamine-treated group.

In the hippocampus and cerebral cortex (Figure 7a,b and Figure S8) of the 6N strain, the scopolamine-treated group exhibited significantly (F (5, 12) = 55.85; *p* < 0.05) higher levels of lipid peroxidation activity (42.76 ± 4.09 nmol/mg protein and 38.04 ± 2.36 nmol/mg protein), compared to the control (11.26 ± 1.17 nmol/mg protein and 8.98 ± 4.82 nmol/mg protein, respectively). This was significantly reversed in the tacrine-treated group (28.59 ± 2.36 nmol/mg protein and 22.29 ± 2.73 nmol/mg protein). In the 6J strain, the scopolamine-treated group exhibited significantly (F (5, 12) = 55.85; *p* < 0.05) higher levels of lipid peroxidation activity (45.91 ± 3.61 nmol/mg protein and 35.68 ± 6.25 nmol/mg protein) compared to the control group (14.02 ± 2.80 nmol/mg protein and 10.47 ± 1.36 nmol/mg protein, respectively), whereas the reversal effect of the tacrine-treated group was not significantly expressed in either the hippocampus or cerebral cortex.

### 2.5. Antioxidant Biomarker Profiles in the Hippocampus and Cerebral Cortex

In the hippocampus of the 6N strain (Figure 7a and Figure S8), the scopolamine-treated group exhibited significantly (F (5, 12) = 8.54, F (5, 12) = 7.06, F (5, 12) = 20.71; *p* < 0.05) lower levels of SOD, CAT, and GPx activities, with values of 33.08 ± 1.50, 545.43 ± 34.18, and 14.03 ± 1.37 U/mg protein, compared to the control group (41.89 ± 1.54, 666.91 ± 28.73, and 32.84 ± 1.71 U/mg protein) respectively. The scopolamine-induced oxidative stress was significantly reversed in the tacrine-treated group (38.19 ± 0.89, 637.96 ± 26.81, and 27.76 ± 2.81 U/mg protein, respectively). Subsequently, a similar antioxidant profile pattern was observed in the cerebral cortex of the 6N strain (Figure 7b). By contrast, while the treated and control groups of the 6J strain varied at both hippocampus and cerebral cortex, these variations were not significant.

### 2.6. Western Blot Analysis of CREB/BDNF in the Hippocampus

**Figure 8:**
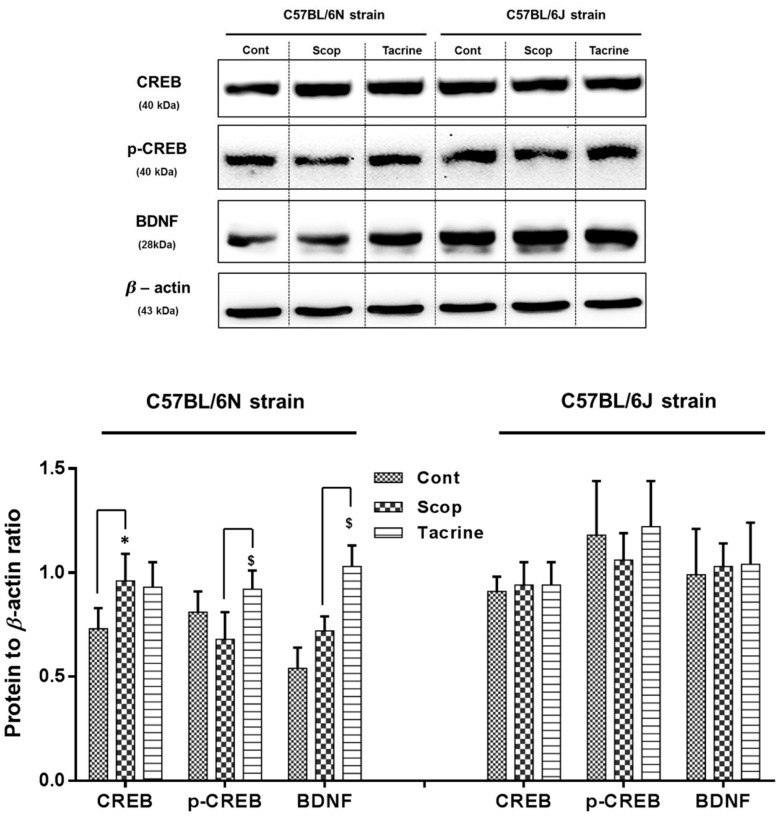
cAMP response-element binding protein (CREB)/brain-derived neurotrophic factor (BDNF) protein markers expression in the hippocampus of scopolamine-induced amnesic models—C57BL/6N and C57BL/6J substrains: Western blotting of CREB, p-CREB, and BDNF protein expression and its quantification relative to β-actin expression. Data are expressed as mean ± SD (*n* = 3). One-way ANOVA-Tukey’s multiple comparison test was performed where, * *p* < 0.05 scopolamine compared with the control group; ^$^ *p* < 0.05 tacrine compared with scopolamine-treated group.

Among the C57BL/6 sub-strains, the scopolamine-treated group of the 6N strain at the hippocampus (Figure 8 and Figures S9–S12) exhibited a significantly higher level of CREB, and a relatively suppressed level of downstream p-CREB and BDNF protein expression compared to the control group, which was significantly reversed in the tacrine-treated group by an approximately 0.5-fold increase. On the other hand, the protein expression levels of the treated and control groups were found to vary less significantly in the 6J strain.

## 3. Discussion

Neurodegenerative disorders are currently in the limelight due to progressive brain malfunction among aging populations in developed countries [26,27]. Progressive neurodegenerative complications result in numerous neurodegenerative diseases, such as Parkinson’s, Alzheimer’s, and Huntington’s. Accumulating evidence suggests that behavioral alterations, cognitive impairment, and cholinergic deficit are the most common parametric events which occur in Alzheimer’s disease [28,29]. The scopolamine-induced amnesia model was adapted in this study to compare various mouse strains for behavioral alterations, cognitive deficits, cholinergic impairment, and associated changes in biochemical and protein expressions. Tacrine is a well-established non-competitive AChE inhibitor, and is utilized as a positive control for the latter part of the study [21,22].

Initially, we assessed the ICR outbred and C57BL/6 inbred mice strains, studying the effects of scopolamine administration on spatial learning and memory deficits. The passive avoidance test (PAT) is one useful method for measuring these effects [30], and our results showed relatively similar patterns of acquisition and retention among both strains: compared to the control group, the scopolamine-treated group consistently failed to acquire the shock memory from day 2 until the retention trial. This was consistent with previous studies in which scopolamine effectively constrained the memory acquisition and retention capacity of animals compared to control groups [31,32]. Spontaneous alternation behavior in the Y-Maze is an effective tool to indicate short-term and working memory of animals [33]. According to previous study reports, scopolamine-inflicted mice effectively suppressed the spontaneous alternation behavior, compared to control groups [31,32,33]. In this study, the C57BL/6 strain showed significant alternation behavior changes between the control and scopolamine-treated groups. However, for as-yet unclear reasons, the ICR strain exhibited non-significant differences between the control and scopolamine-treated groups. AChE plays a vital role in the hydrolysis of the ACh neurotransmitter, which was found to be decreased in hippocampal and cortical levels of AD patients, resulting in cognitive impairment [34,35]. This study analyzed AChE levels in the hippocampi of both strains. In the hippocampus of the C57BL/6 strain, significant differences in AChE activity were observed between the scopolamine-treated and control groups, whereas the differences were found to be insignificant in the ICR strain. Recent reports suggest a possible link between cholinergic impairment, mediated behavioral alteration, and motor functional deficits [36,37]. Accordingly, we propose that the interventional anomaly exhibited in the Y-Maze alternation behavior of the ICR strains may have occurred due to insignificant neuronal impairment by scopolamine in the cholinergic system, as attributed to strain and dose-dependent deficiencies in earlier studies [38,39]. For further confirmation, as reported in earlier studies [40,41], the expression of proteins associated with amnesic neuro-inflammation such as iNOS, COX-2, and IκBα/p-IκBα has also been evaluated at the hippocampus. The scopolamine-administered group in the C57BL/6 strain exhibited significant elevation of inflammatory protein expression compared to the control group, whereas no significant difference was noted in the ICR strain. This most likely suggests that the dose of scopolamine administered (2 mg/kg of animal body weight) induces significantly lesser amnesic effects in the outbred ICR strain compared to the inbred C57BL/6 strain.

Subsequently, we extended our study to compare the scopolamine-induced amnesic effects between two sub-strains of C57BL/6: 6N and 6J. In our behavioral studies (PAT and Y-Maze), the 6N strain exhibited significant scopolamine-inflicted behavioral deficits and reversal effects in the tacrine-treated group, whereas these differences were relatively less significant in the 6J strain. In accordance, AChE activity in both the hippocampus and cerebral cortex also differed significantly between the treated groups of the 6N strain, compared to the 6J strain. This was in correlation with our previous discussion of cholinergic impairment, mediated behavioral alteration, and motor functional deficits. Moreover, earlier research has claimed that avoidance behavior demands higher cognitive functions involving the hippocampus and cortex [42,43]. These facts collectively suggest that scopolamine administered at the proposed dose effectually induced cholinergic impairment in both hippocampus and cerebral cortex of the 6N strain, and therefore significant behavioral alterations, but not in the 6J strain. Considerable research data also suggests that in most neurodegenerative disorders (including scopolamine-induced amnesia), impairment is comprised of significant oxidative stress and neuronal inflammation [44,45,46]. Oxidative stress occurs due to the accumulation or insufficient degradation of reactive oxygen species (ROS) in brain cells, which leads to DNA, lipid, and protein impairment [47,48]. This stress-mediated injury triggers astrocytes and microglia-facilitated inflammatory reaction [49,50]. This interplay leads to neuronal cell death and mediated neurodegenerative complications. Accordingly, in this study, the scopolamine-treated groups of the 6N strain exhibited substantial oxidative stress and inflammatory protein expression in the hippocampus and cerebral cortex compared to the control group, which was effectually ameliorated in the tacrine-treated groups. However, the 6J strain exhibited non-significant differences in oxidative stress and inflammatory alterations among the various treated groups. These findings were comparable with previous research, as 6J and 6N mice exhibited different courses of inflammation, apoptosis, and ROS-induced DNA damage in a hypoxic–ischemic brain injury model [51]. A likely reason behind this variance between the strains might be due to a nicotinamide nucleotide transhydrogenase (Nnt) mutation. Nnt is a mitochondrial protein which plays a vital role in the reduction of nicotinamide adenine dinucleotide phosphate (NADP^+^), which in turn triggers a NADPH-mediated ROS elimination process [52]. Lack of Nnt results in elevated ROS levels and oxidative stress [52,53]. Thus, lack of Nnt protein in the 6J strain is likely responsible for the oxidative stress and inflammatory response disparities among the sub-strains. Furthermore, we evaluated the modulations of CREB/BDNF protein expression in the hippocampus. BDNF is a neurotrophin that is known to play a beneficial role in enhancing learning ability and neurogenesis via phospho-CREB signaling [54], whereas the transcription factor CREB is responsible for memory and synaptic plasticity in the central nervous system [55]. Accumulated evidence supports the constructive role of the CREB/BDNF pathway in enhancing memory and cognition, and disturbance of phosphorylated CREB/BDNF levels in the hippocampal region leading to the progression of neurodegenerative diseases such as Alzheimer’s and Parkinson’s [55,56]. In addition, previous reports indicate that scopolamine-inflicted mice show reduced levels of p-CREB/BDNF levels in the hippocampus [57,58]. Accordingly, in our study, the scopolamine-inflicted group of the 6N strain showed a substantial reduction in hippocampal p-CREB/BDNF levels compared to the control group, which was effectively alleviated by tacrine administration. In contrast, the p-CREB/BDNF levels did not differ among the treated groups of the 6J strain. Meanwhile, a recent study has associated CREB/BDNF pathway proteins with neuroprotective signaling against ROS-mediated cell death [59]. This suggests that a lack of Nnt-associated oxidative stress impairment in the 6J strain might possibly be related to the slightly non-significant p-CREB/BDNF protein expression among the treated groups. However, further studies with a higher number of “*n*” animals can possibly provide a clear picture on the cognitive, behavioral and biochemical fluctuations among the evaluated strains/sub-strains. Additionally, the causal relationship between oxidative stress and CREB/BDNF protein needs further clarification.

## 4. Materials and Methods

### 4.1. Reagents

Scopolamine hydrochloride and tacrine hydrochloride hydrate (THA) were purchased from Sigma (St. Louis, MO, USA).

### 4.2. Animals and Treatment

ICR outbred and C57BL/6 inbred male mouse strains (each strain *n* = 14; age, 8–9 weeks; weight, 24–27 g) were obtained from the Samtaco Inc. (Osan, Korea). C57BL/6N and C57BL/6J male mouse strains (each strain *n* = 21; age, 8–9 weeks; weight, 24–27 g) were obtained from the Deahan Bio Link (Eumseong, Korea). All the mice strains were housed in a controlled environment (23 ± 1 °C; 50 ± 5% humidity; 12 h dark–light cycle) and allowed water and food ad libitum. After a week of acclimatization, animals were randomly divided into experimental groups.

#### 4.2.1. ICR and C57BL/6 Comparison Study

The grouping of this study includes control (*n* = 7, 0.9% saline, i.p., single dose/day) and scopolamine 2 mg/kg (*n* = 7, scopolamine in 0.9% saline i.p., single dose/day). From day 1 to day 3, saline and scopolamine were administered to the respective groups 5 min prior to the start of the passive avoidance acquisition task. On day 4, the passive avoidance retention task was performed.

#### 4.2.2. C57BL/6N and C57BL/6J Comparison Study

The grouping of C57BL/6N (6N) and C57BL/6J (6J) comparison study includes control (*n* = 7), scopolamine 2 mg/kg (*n* = 7), and tacrine 10 mg/kg (*n* = 7). All animals were orally administered with distilled water (control and scopolamine) or tacrine for seven days prior to scopolamine injection. On day 8, scopolamine was dissolved in 0.9% saline (2 mg/kg) and—with the exception of the control group—administered i.p. 30 min before the passive avoidance acquisition task. In both studies, following the acquisition day, 30 min after the passive avoidance retention task, the animals were subjected to Y-Maze spontaneous alternation. All the experiments were performed in accordance with the Principles of Laboratory Animal Care (NIH publication no. 85–23, revised 1985) and approved by Konkuk University Institutional Animal Care and Use Committee (KU17068; 26 April 2017).

### 4.3. Behavioral Studies

#### 4.3.1. Step-Through Passive Avoidance Test

Experimental procedures were performed using the Gemini Active and Passive Avoidance System (San Diego Instruments, San Diego, CA, USA), associated with a computerized system as described earlier [60], with slight modification. The instrument comprises a light and a dark partition separated by a guillotine door. Though both of the partitions look alike, the floor of the dark compartment can induce an electric shock up to 0.5 mA, and is computer-controlled. Each animal was habituated with the behavioral instrument for 2–3 min before the acquisition session, without shock treatment. On the day of acquisition, the animals were placed in the lit compartment with their heads facing the wall. After an acclimatization period of 30 s, the guillotine door was programmed to open and the animal was subjected to a trial of 270 s. Upon entering the dark compartment, the door was programmed to shut and the animal was punished with single low-intensity foot shock of 0.5 mA for 5 s. The time latencies (LTs) at which the animals stepped into the dark compartment were recorded by the computer. Twenty-four hours after the acquisition trial, the retention trial was conducted with the same protocol, except that no shock was delivered when the animal entered the dark compartment. The criterion for learning was the increase in LT from acquisition trial to retention trial.

#### 4.3.2. Spontaneous Alternation Performance (Y-Maze Test)

A single session of Y-Maze was used to record the spontaneous alternation behavior to determine the instant memory functioning and exploratory behavior of the animals. The Y-Maze comprises three equal arms in a “Y” shape, with dimensions of 40 × 12 × 30 per arm. The experimental procedure was performed as described previously [60]. In brief, each animal was placed in the maze, at the end of one arm, and allowed to explore freely throughout the maze for 8 min. The number of arm entries were noted. One arm entry was considered when the hind paws of the animal were completely placed inside the arm, and the series of arm entries were documented visually by a person blinded to the experimental groups. Alternation was defined as consecutive entries into the three arms, on overlapping triplet sets. The percentage of alternation can be calculated using the following formula:

Percentage alternation = [(number of alternations)/(total number of arm entries − 2)] × 100

### 4.4. Tissue Procurement and Protein Quantification

One hour after the Y-Maze test, all animals were sacrificed by decapitation under anesthesia. The whole brain was carefully removed by skull incision and washed twice with cold normal saline solution, followed by procurement of hippocampus and cerebral cortex as described earlier [60]. A portion of obtained tissue samples (*n* = 4) were homogenized using (10% *w*/*v*) cold phosphate-buffered saline (PBS, 0.1 M, pH 7.4) and the supernatants were stored at −70 °C for measurement of AChE. The another portion of tissue samples (*n* = 4) from individual groups were pooled and subjected for the analysis of malondialdehyde (MDA) level, superoxide dismutase (SOD), catalase (CAT), and glutathione peroxidase (GPx) activity. The remaining animals tissue samples (*n* = 3) were homogenized using RIPA lysis buffer (Millipore, Billerica, MA, USA) with protease inhibitor cocktail (Roche, Mannheim, Germany) and the supernatants were stored at −70 °C for Western blot analysis. The protein level of the homogenates was quantified using a Bio-Rad DC Protein Assay kit, according to the manufacturer’s protocol, and normalized for further analysis.

### 4.5. AChE Activity

Acetylcholinesterase (AChE) catalyzes the hydrolysis of the neurotransmitter acetylcholine, which is an active participant in neuro-signaling. AChE activity in the hippocampus and cerebral cortex homogenates was determined using a Biochain kit (Biochain, San Francisco, CA, USA), according to the manufacturer’s protocols, as described previously [60]. Briefly, the thiocholine produced by the action of AChE forms a yellow color with 5,5′-dithiobis (2-nitrobenzoic acid), which was quantified at 410 nm using an ultra-violet spectrophotometer.

### 4.6. Lipid Peroxidation

The oxidative stress can be assessed by quantification of lipid peroxidation in the tissue. Malondialdehyde (MDA) is a marker of lipid peroxidation, and was measured in the tissue homogenate using lipid peroxidation colorimetric/fluorometric assay kit (BioVision, Milpitas, CA, USA) according to the manufacturer’s protocol. In brief, the MDA in the sample reacts with supplied thiobarbituric acid (TBA) to form MDA–TBA adducts, which was quantified colorimetrically at 532 nm. MDA levels in the sample homogenates were expressed as nanomoles of MDA per milligram of protein.

### 4.7. SOD, CAT, and GPx Analysis

SOD, CAT, and GPx are well-known antioxidant biomarkers responsible for the defense system, used to counteract the tissue oxidative stress. SOD and CAT activity in tissue homogenates were evaluated using an Oxiselect superoxide dismutase activity assay kit (Cell Biolabs, San Diego, CA, USA). The SOD assay utilizes a xanthine/xanthine oxidase system to generate superoxide anions, which reduce a chromogen to a water-soluble formazan dye. The activity of SOD in the tissue homogenate was determined as the inhibition of chromogen reduction, which can be quantified colorimetrically at 490 nm. The CAT assay utilizes the action of CAT in the decomposition of hydrogen peroxide. The activity of CAT in the tissue homogenate was determined by the quenching of hydrogen peroxide, and quantification of the residual hydrogen peroxide using a coupling reaction measured colorimetrically at 520 nm. The GPx activity in tissue homogenates was evaluated using a glutathione peroxidase activity colorimetric assay kit (BioVision, Milpitas, CA, USA), according to the manufacturer’s protocol. Briefly, GPx in the tissue homogenate reduces cumene hydroperoxide, while oxidizing glutathione (GSH) to oxidized glutathione (GSSG). The generated GSSG is reduced to GSH with consumption of NADPH by glutathione reductase (GR). The decrease of NADPH is proportional to GPx activity, and was measured colorimetrically at 340 nm.

### 4.8. Western Blot Analysis

Tissue homogenate was prepared in RIPA lysis buffer (Millipore, Billerica, MA, USA) with protease inhibitor cocktail (Roche, Mannheim, Germany). After protein normalization, samples (20 μg protein per lane) were separated in 10% SDS-polyacrylamide gels and transferred electrophoretically to PVDF membrane (Millipore, Bedford, MA, USA). The membranes were blocked with 5% bovine serum albumin (BSA) in Tris buffered saline (TBS, pH 7.4) for 1 h, then incubated overnight at 4 °C with primary antibodies: anti-iNOS (1:2000), anti-IκB-α (1:1000) and anti-phospho-IκB-α (1:1000) (Cell Signaling Technology); anti-COX-2 (1:2000), anti-β-actin (1:1000) (Santa Cruz Biotechnology); anti-CREB (1:5000), anti-phospho-CREB (1:5000), anti-BDNF (1:1000) (Abcam), followed by incubation for 1 h with horseradish peroxidase-conjugated secondary antibodies (1:10,000) (Cell Signaling). The optical images of the antibody-specific bands were captured by a Davinch-Chemi & Fluoro Imaging System (Seoul, Korea), and their relative band densities were analyzed by Image J software (version-1.47; Bethesda, MD, USA).

### 4.9. Statistical Analysis

All data were analyzed using Graph Pad Prism, version 5.01 (La Jolla, CA, USA). They were expressed as mean ± SD analyzed by two-way ANOVA for behavioral studies and one-way ANOVA for other parameters both analyzed with Tukey’s multiple comparison test. In the present study, we compared the control group with the scopolamine-administered group and/or the tacrine-treated group. In all cases, probability values of *p* < 0.05 were considered statistically significant.

## 5. Conclusions

Overall, we conclude that the inbred C57BL6 mouse strain better reveals scopolamine-induced learning and memory deficits, cholinergic impairment, and neuro-inflammation compared to the outbred ICR strain. Furthermore, among the two C57BL6 sub-strains, the 6N strain was found to be more effective than the 6J strain at exhibiting scopolamine-inflicted modulations in behavior, cognition, inflammation, and oxidative stress, possibly due to lack of Nnt protein. However, further comprehensive investigations with a higher “*n*” values are required to understand the reasons behind strain difference, and also the potential link between events of neuronal impairment. This may pave the way for future research to study the underlying mechanisms involved in scopolamine-induced amnesia, and to aid the screening of novel drug candidates to combat various neurodegenerative complications.

## Supplementary Materials

Supplementary materials can be found at www.mdpi.com/1422-0067/18/8/1735/s1.

## Abbreviations

AD	Alzheimer’s disease
AChE	Acetylcholine Esterase
BDNF	Brain-derived neurotrophic factor
BSA	Bovine serum albumin
COX-2	Cyclooxygenase-2
CAT	Catalase
CREB	cAMP response-element binding protein
GPx	Glutathione peroxidase
GSH	Glutathione
GSSG	Oxidized glutathione
GR	Glutathione reductase
IκBα	Nuclear factor of κ inhibitor
iNOS	Inducible nitric oxide synthase
LT	Time latencies
MDA	Malondialdehyde
NADP^+^	Nicotinamide adenine dinucleotide phosphate
Nnt	Nicotinamide nucleotide transhydrogenase
PAT	Passive avoidance test
p-IκBα	Phosphorylated-nuclear factor of κ inhibitor
PBS	Phosphate-buffered saline
PVDF	Polyvinylidene fluoride
ROS	Reactive oxygen species
SOD	Superoxide dismutase
TBA	Thiobarbituric acid
THA	Tacrine hydrochloride hydrate
