# Supplementary material for: Comparative Studies on Behavioral, Cognitive and Biomolecular Profiling of ICR, C57BL/6 and Its Sub-Strains Suitable for Scopolamine-Induced Amnesic Models
Source: Int J Mol Sci. 2017 Aug 9;18(8):1735. doi: 10.3390/ijms18081735 (PMC5578125; doi:10.3390/ijms18081735)
Supplement: Supplementary file 1 [file ijms-18-01735-s001.pdf]

Comparative Studies on Behavioral, Cognitive and Biomolecular Profiling of ICR, C57BL/6 and Its Sub-Strains Suitable for Scopolamine-Induced Amnesic Models

Govindarajan Karthivashan [1], Shin-Young Park [1], Joon-Soo Kim [1], Duk-Yeon Cho [1], Palanivel Ganesan [1,2] and Dong-Kug Choi [1,2,*]

Supplement Figures:

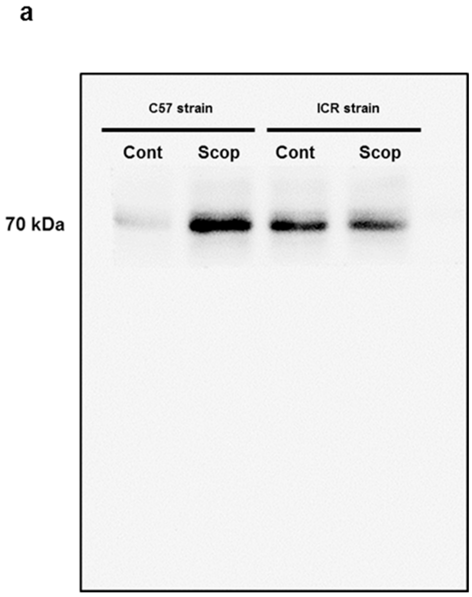

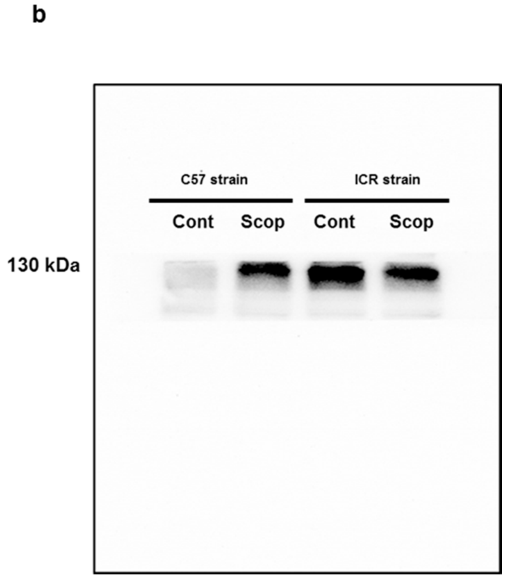

Figure.S1. ICR and C57BL/6 strains – a) COX-2 (70 kDa); b) iNOS (130 kDa).

a

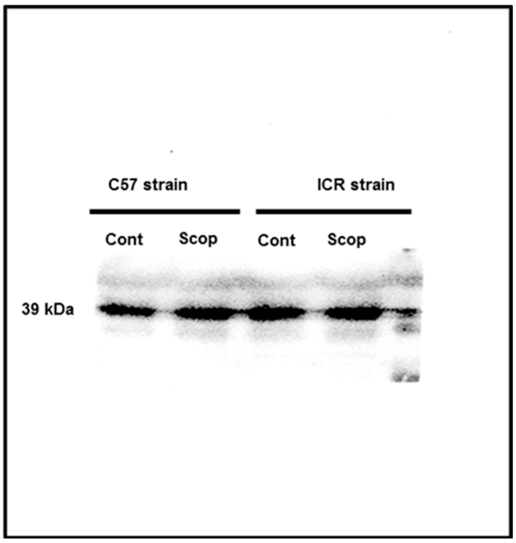

b

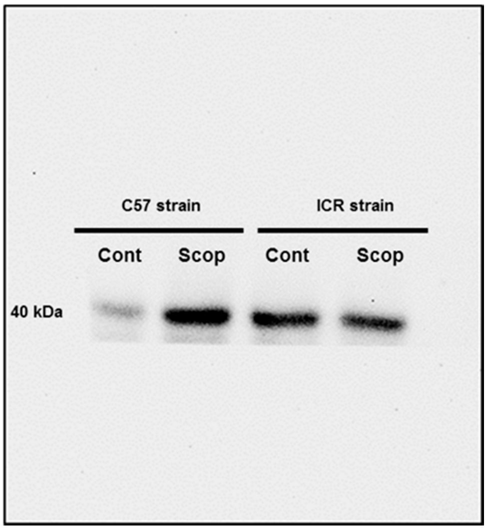

Figure.S2. ICR and C57BL/6 strains – a) Ikβα (39 kDa); b) p-Ikβα (40 kDa).

b

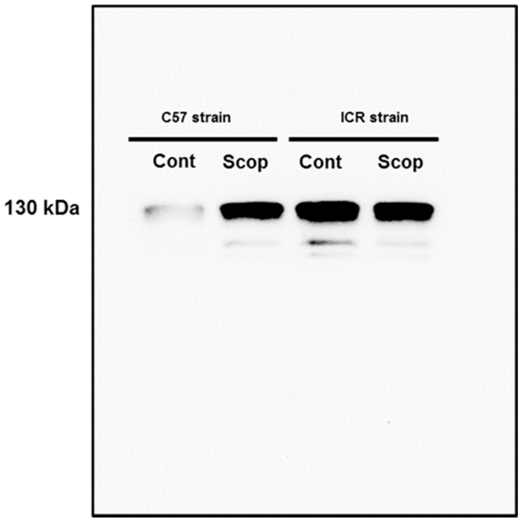

Figure.S3. ICR and C57BL/6 strains – a) COX-2 (70 kDa); b) iNOS (130 kDa).

a

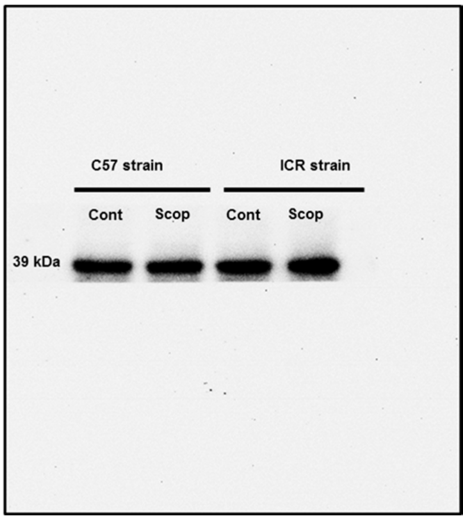

b

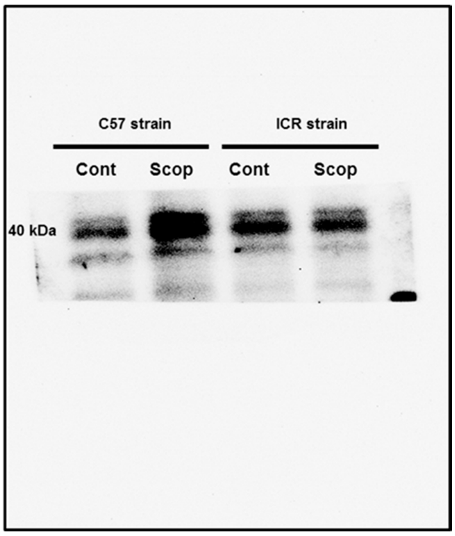

Figure.S4. ICR and C57BL/6 strains – a) Ikβα (39 kDa); b) p-Ikβα (40 kDa).

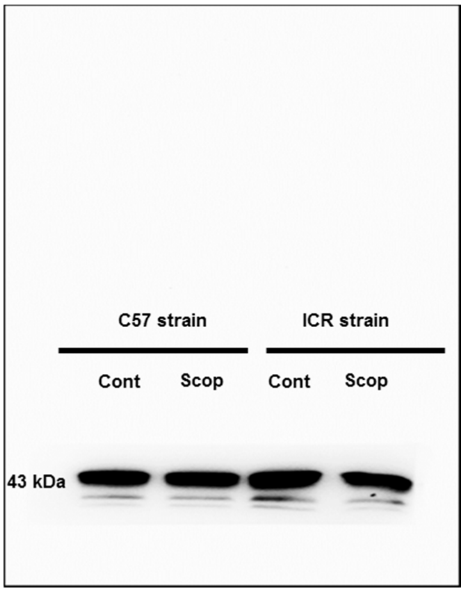

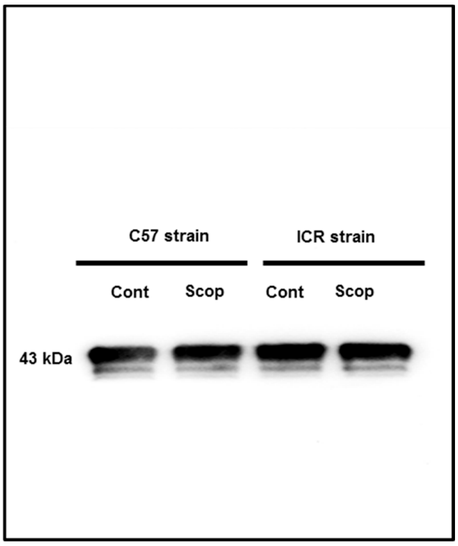

Figure.S5. ICR and C57BL/6 strains – β-actin (43 kDa)

a

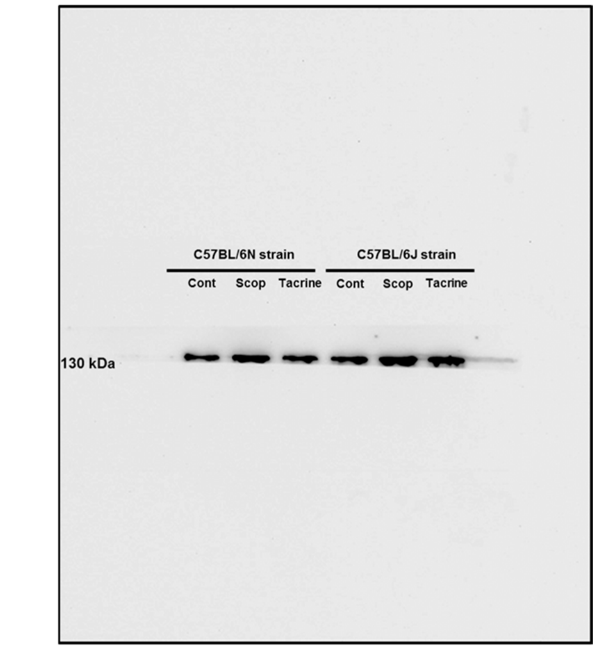

b

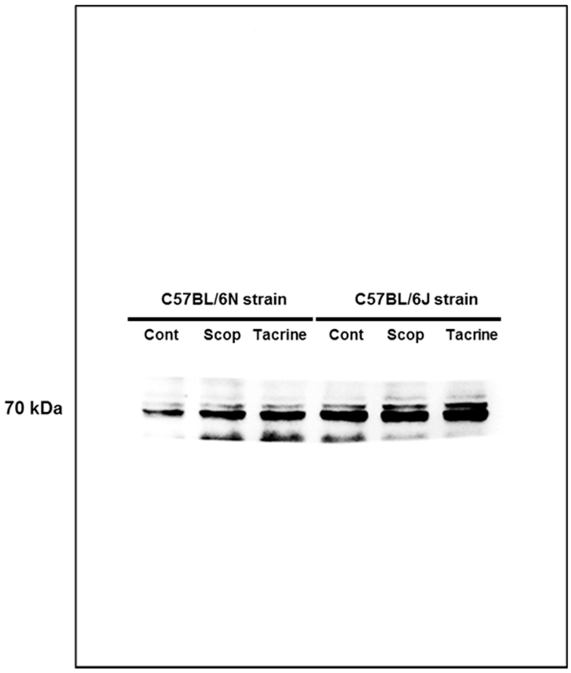

Figure.S6. C57BL/6N and C57BL/6J strains – a) iNOS (130 kDa); b) COX-2 (70 kDa)

a

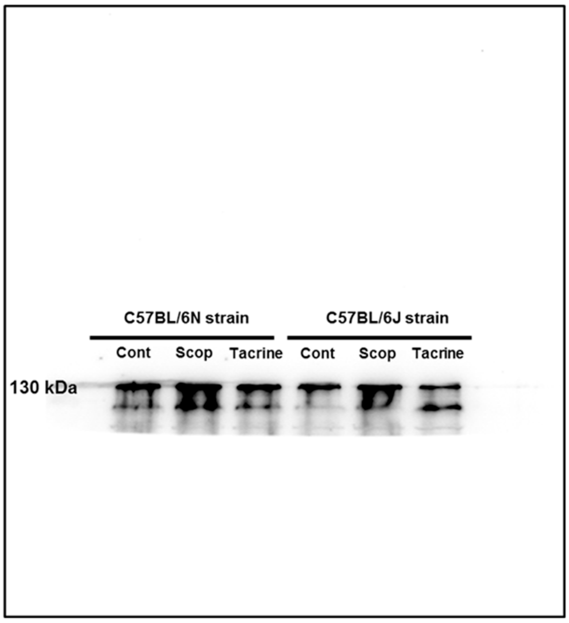

b

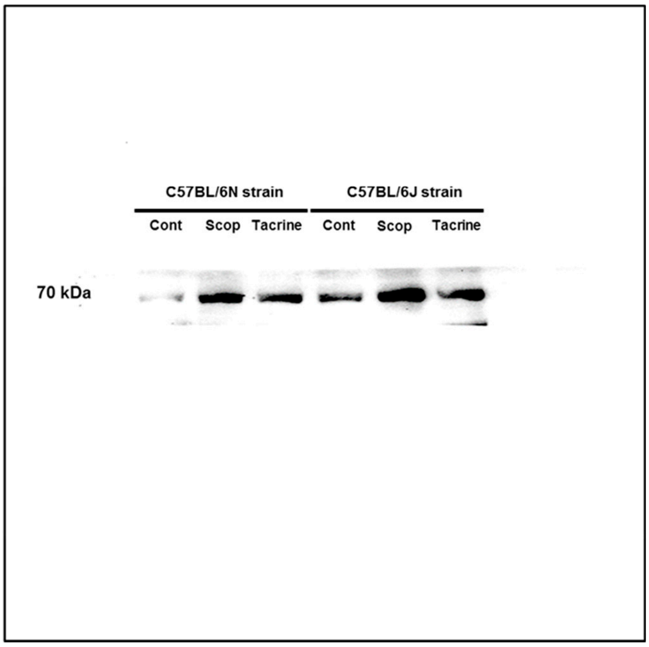

Figure.S7. C57BL/6N and C57BL/6J strains – a) iNOS (130 kDa); b) COX-2 (70 kDa)

a

MDA std nmol	1	2	N				J			
0	0.047	0.048	C- Hip	0.078	0.079	0.079	C- Hip	0.079	0.081	0.079
1	0.458	0.445	C-C.C	0.076	0.077	0.080	C-C.C	0.078	0.078	0.079
2	0.724	0.711	S- Hip	0.093	0.093	0.090	S- Hip	0.092	0.093	0.095
3	1.117	1.124	S-C.C	0.090	0.091	0.089	S-C.C	0.092	0.087	0.088
4	1.424	1.463	T- Hip	0.086	0.087	0.085	T- Hip	0.091	0.087	0.090
5	1.711	1.723	T-C.C	0.084	0.084	0.082	T-C.C	0.087	0.085	0.085
			N-Hipp		J-Hipp		N-C.C		J-C.C	
Calculated values		Con	11.26	1.17	14.02	2.80	8.98	4.82	10.47	1.36
		Scop	42.76	4.09	45.91	3.61	38.04	2.36	35.68	6.25
		Tac	28.59	2.36	36.46	4.92	22.29	2.73	27.80	2.73

b

SOD std					N				J	
1000	0.193	0.140	C- Hip	0.436	0.429	0.431	C- Hip	0.428	0.439	0.437
500	0.227	0.223	C-C.C	0.437	0.441	0.439	C-C.C	0.431	0.449	0.436
250	0.281	0.282	S- Hip	0.454	0.457	0.447	S- Hip	0.437	0.446	0.442
125	0.285	0.285	S-C.C	0.465	0.468	0.457	S-C.C	0.442	0.445	0.439
62.5	0.047	0.311	T- Hip	0.439	0.440	0.443	T- Hip	0.441	0.443	0.437
31.25	0.417	0.464	T-C.C	0.457	0.464	0.455	T-C.C	0.444	0.438	0.447
			N-Hipp		J-Hipp		N-C.C		J-C.C	
Calculated values		Con	41.89	1.54	40.75	2.50	38.90	0.85	39.04	3.96
		Scop	33.08	1.50	37.77	1.92	28.56	2.41	37.62	1.28
		Tac	38.19	0.89	38.33	1.30	30.52	2.01	37.20	1.95

c

CAT std			N				J			
100	0.175	0.173	C- Hip	0.944	0.972	0.946	C- Hip	1.012	0.992	0.984
50	0.441	0.462	C-C.C	1.009	0.994	0.980	C-C.C	1.012	1.027	1.002
25	0.735	1.006	S- Hip	1.039	1.003	1.034	S- Hip	1.032	1.009	1.002
12.5	1.181	1.181	S-C.C	1.035	1.058	1.064	S-C.C	1.044	1.020	1.046
6.25	1.268	1.262	T- Hip	0.955	0.990	0.968	T- Hip	1.027	0.991	0.990
3.125	1.318	1.298	T-C.C	1.010	1.008	1.038	T-C.C	1.021	1.036	1.037
1.5625	1.323	1.321								
0	1.354	1.394								
			N-Hipp		J-Hipp		N-C.C		J-C.C	
		Con	666.91	28.73	595.38	24.56	598.22	31.34	565.29	34.59
Calculated values		Scop	545.43	34.18	564.16	28.40	499.45	26.80	526.13	21.59
		Tac	637.96	26.81	584.03	36.45	556.78	31.08	535.21	15.47

d

GPx (10 min)	N				J			
	C- Hip	1.085	1.064	0.532	C- Hip	0.536	0.522	0.472
	C-C.C	1.033	1.012	0.486	C-C.C	0.519	0.486	0.465
	S- Hip	1.085	1.064	0.505	S- Hip	0.512	0.503	0.530
	S-C.C	1.058	1.037	0.506	S-C.C	0.523	0.488	0.468
	T- Hip	1.068	1.047	0.495	T- Hip	0.504	0.466	0.446
	T-C.C	1.078	1.057	0.518	T-C.C	0.531	0.537	0.534
GPx (20 min)	N				J			
	C- Hip	0.871	0.865	0.328	C- Hip	0.335	0.331	0.301
	C-C.C	0.843	0.821	0.287	C-C.C	0.339	0.289	0.276
	S- Hip	0.958	0.949	0.382	S- Hip	0.369	0.333	0.372
	S-C.C	0.932	0.901	0.369	S-C.C	0.348	0.315	0.295
	T- Hip	0.872	0.865	0.324	T- Hip	0.340	0.308	0.271
	T-C.C	0.911	0.891	0.366	T-C.C	0.359	0.347	0.356
Calculated values								
	N-Hipp		J-Hipp		N-C.C		J-C.C	
Con	32.84	1.71	28.81	3.42	30.07	1.10	29.03	1.90
Scop	14.03	1.37	21.94	3.03	16.57	1.36	25.67	0.26
Tac	27.76	2.81	23.88	1.93	22.99	1.88	27.09	2.05

Figure S8: Raw absorbance and corresponding calculated values of the a) lipid peroxidation (malondialdehyde, MDA); b) superoxide dismutase (SOD); c) catalase (CAT); d) glutathione peroxidase (GPx) levels in the hippocampus (Hipp) and cerebral cortex (C.C) of scopolamine-induced amnesic models—C57BL/6N and C57BL/6J sub-strains. Data are expressed as mean ± SD (n = 4; pooled biological replications).

a

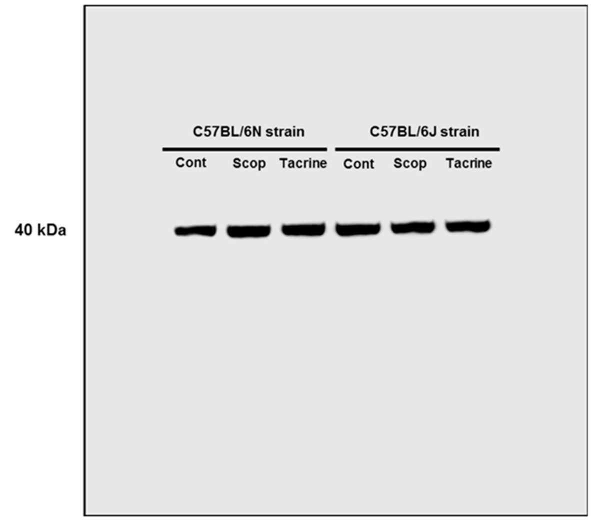

b

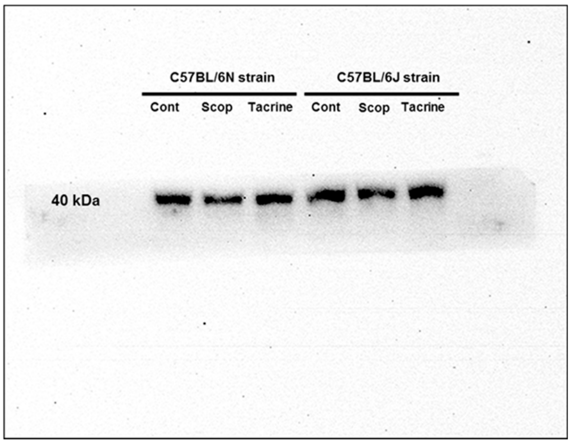

Figure.S9. C57BL/6N and C57BL/6J strains – a) CREB (40 kDa); p-CREB (40 kDa)

a

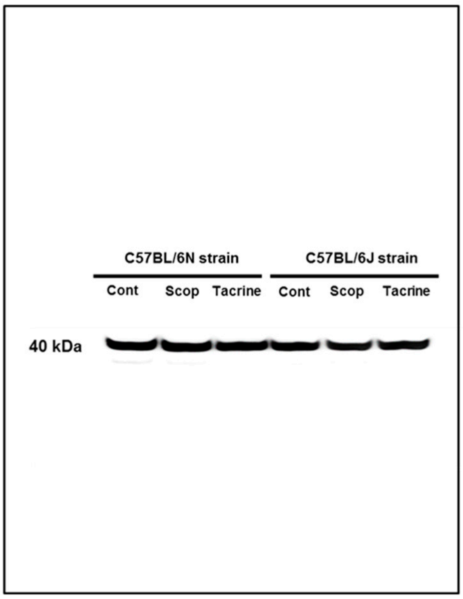

b

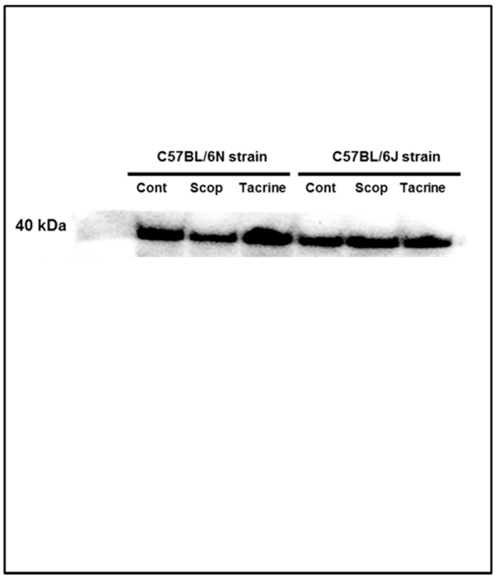

Figure.S10. C57BL/6N and C57BL/6J strains – a) CREB (40 kDa); p-CREB (40 kDa)

a

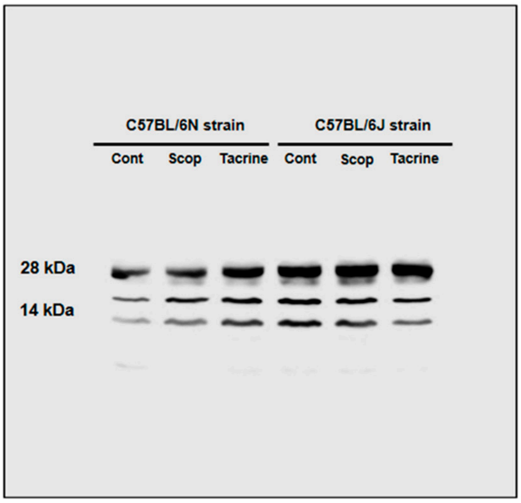

b

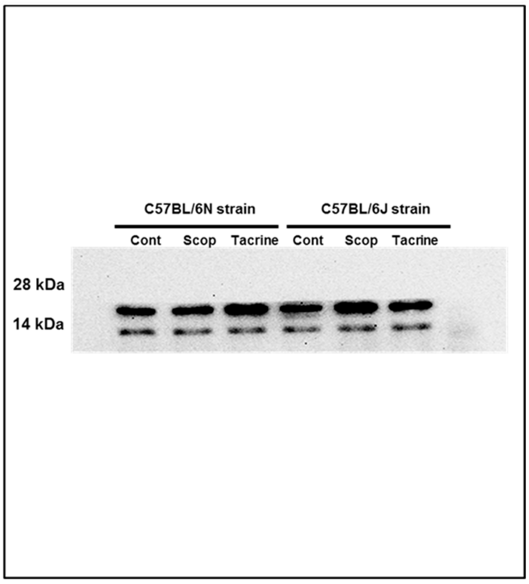

Figure.S11. C57BL/6N and C57BL/6J strains – BDNF (28 kDa); below bands represents monomer at approximately 14 kDa.

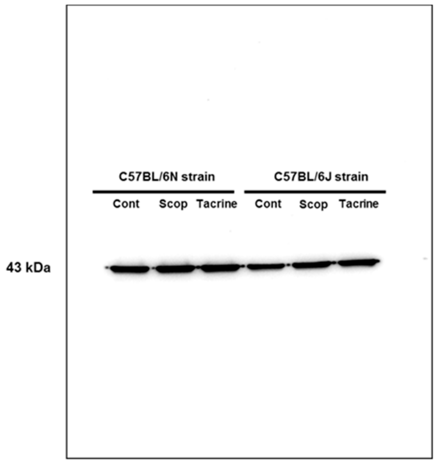

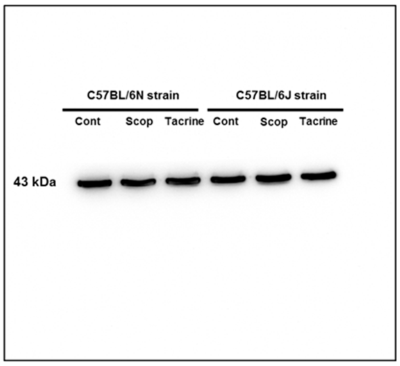

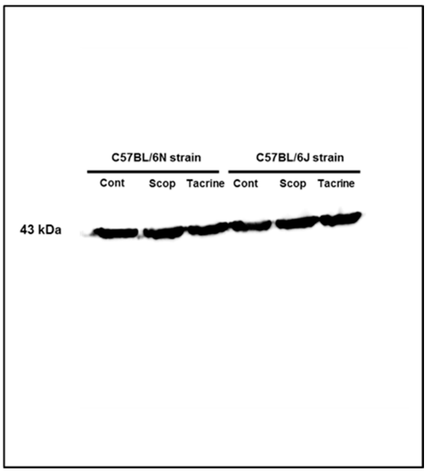

Figure.S12. C57BL/6N and C57BL/6J strains - β-actin (43 kDa).
